# Heterozygous variants of *NOD2*, *IL10RA*, *PLA2G6* and *COL7A1* correlate with Crohn's disease

**DOI:** 10.1016/j.heliyon.2023.e22968

**Published:** 2023-11-30

**Authors:** Qiang Zhang, Xizi Wang, Juan Zheng, Qiang Lü, Rongrong Li, Xiaodong Jia, Mingliang Gu

**Affiliations:** Joint Laboratory for Translational Medicine Research, Liaocheng People's Hospital, Liaocheng, China

**Keywords:** Crohn's disease, Whole exome sequencing, *NOD2*, *IL10RA*, *PLA2G6*, *COL7A1*

## Abstract

To identify candidate pathogenic genes of early-stage Crohn's disease (CD) and predict potential roles of genetic factors in CD, we performed whole exome sequencing on a child with early-stage Crohn's disease (CD) and her parents (core family), found that the patient carried heterozygous variants of 4 genes: *NOD2* c. 2257 C > T, *IL1*0RA c. 301 C > T, *PLA2G6* c. 2029 C > T, *COL7A1* c. 3190 G > A. Heterozygous variants of *NOD2*, *IL1*0RA, *PLA2G6* and *COL7A1*, intestinal inflammatory response is triggered, normal intestinal wall tissue damage, leading to CD phenotype.

## 1 Introduction

Crohn's disease (CD) is an inflammatory bowel disease (IBD) involving any part of the gastrointestinal tract [1]. CD is characterized by intermittent, progressive and destructive transmural inflammation. Patients often present abdominal pain, diarrhea, and repeated anal lesions with delayed healing [2,3]. Since the 21st century, the incidence of IBD has been increased worldwide, with the highest mainly in North America, Northern Europe and Western Europe [4]. Although the prevalence of IBD in Asia is relatively lower than that in western countries, the incidence of CD has been increased rapidly [1]. In China, IBD has become a common disease, with its incidence exhibiting a gradient distribution from the south to north, and from the east to west [5,6].

Genetic susceptibility and host reactions (immune regulation and intestinal flora) contribute to CD [7,8]. Genome-wide association analysis revealed more than 200 loci associated with CD risk, which are mainly located in regulatory regions [9]. For example, *NOD2*, *ATG16L1*, *IRGM*, *LRRK2* and *XBP1* genes are associated with susceptibility to CD. These variants are proposed to cause abnormal secretion activity of Paneth cells, endoplasmic reticulum stress signals that may trigger pathological unfolded protein response (UPR), intestinal barrier dysfunction, and intestinal inflammatory response [[10], [11], [12], [13], [14], [15]]. Abnormalities of STAT3 and NF-κB pathways lead to intestinal barrier dysfunction, mediating entry of luminal contents into lamina propria, activation of innate and adaptive immunity, and production of pro-inflammatory factors that perpetuate intestinal inflammation [16]. *IL-10* and *IL-10R* genetic variants are associated with early-onset IBD and Mendelian inheritance of high intestinal wall permeability [17]. *IL23R* gene is associated with predisposition to CD [18]. In addition, in genetically susceptible hosts, decreased diversity of host intestinal flora correlates with the development of CD [1]. In individuals, genetic factors interact with intestinal flora or pathogens to trigger a sustained over-active immune response in the intestinal tract, resulting in intestinal tissue damage and aggravating intestinal barrier dysfunction [19]. Although extensive studies have been conducted on CD, its etiology and pathogenesis are not fully understood. CD remains an incurable and complex disease due to limited prevention strategies and treatment approaches.

We performed whole exome sequencing on a neonatal patient with severe CD and her parents. Candidate pathogenic genes of the neonatal patient were screened and identified using databases of CD pathogenic genes and colorectal tissue-specific genes. Through gene function annotation, pathway analysis and protein structure prediction, biological functions affected by genetic variants were explored to reveal the genetic mechanisms underlying severe CD phenotypes.

## 2 Material and methods

### 2.1 Research objects

The patient, female, was born at full term in 2010. She was admitted to Beijing Children's Hospital due to diarrhea and anal fissure with perianal abscess more than ten days after birth. Her body temperature was over 38° Celsius and c-reactive protein level was high. After hospitalization, antibiotics were administered. No improvement was observed in perianal abscess ulceration accompanied by inflammation, or anal fissure wound. At age 1, Ileostomy and Repair of intestinal perforation were performed. At age 2, high fever, dehydration, anemia and hypoproteinemia repeatedly occur. Colonoscopy identified scattered white scars and patchy hyperemia of the small intestine mucosa, and pebbly nodules of the sigmoid colon mucosa with hyperemia. Pathological findings revealed (1) mild to moderate chronic inflammation of superficial mucosal tissues of the small intestine, accompanied by mild acute inflammation and lymphoid hyperplasia; (2) severe chronic inflammation of sigmoid colon, accompanied by moderate, acute inflammation and lymphoid hyperplasia; (3) congestion and edema of ileocecal intestinal mucosa, partial mucosal degeneration and exudation, scattered inflammatory cell infiltration of intestinal wall, accompanied by mesenchymal hyperemia. At age 3, “total colectomy”, “rectum scaphoid fossa fistula” and “jejunal anostomy” were performed. The overall postoperative condition was improved, with normal growth and development. However, fever, infection, dehydration, diarrhea and infections frequently occurred. After symptomatic treatment, her symptoms were gradually relieved. At age 5, pathology revealed (1) mild edema of rectal mucosa with infiltration of chronic inflammatory cells (lymphocytes, plasma cells, and eosinophils), (2) no reduction of goblet cells, (3) extensive Paneth cell metaplasia, (4) mild conjunctival tissue hyperplasia of lamina propria, and (5) no granulomatous lesions.

Her parents reported a family history of immune diseases of varying degrees. Her father suffered from mild asthma as a child, and recovered after puberty. Her mother suffered from mild dermatitis and her grandmother suffered from mild asthma. None of the parents had a family history of IBD. In 2017, blood samples of the patient and her parents were collected and examined at the Joint Laboratory of Translational Medicine Research, Liaocheng People's Hospital. This study was approved by the Ethics Committee of Liaocheng People's Hospital. All subjects signed informed consent, and the child's written informed consent was provided by her legal guardians.

### 2.2 Whole exome sequencing

Firstly, genomic DNA was extracted from whole blood by QIAamp DNA blood MIDI Kit (Qiagen, Germany), and quality control was performed by nanodrop (Thermo Fisher Scientific, USA), OD 260/280 ratio of 1.8–2.0 [20].

Secondly, DNA was interrupted into ∼200 bp fragments with Covaris S220. Breaking parameters were setup as follows: Duty factor 10 %; Peak Incident Power 175; Cycles per Burst 200; Treatment time 360s; and Bath Temperature 4°C-8 °C. Aglinet 2100 was used for quality control.

Thirdly, Agilent Sureselect DNA Targeting Sequence Capture Kit was applied for library preparation as follows: (1) end repair was performed on fragmented DNA. The A was added to 3′ end, and gap was connected with adapters. After each step, AMPure XP beads was used for purification. (2) Polymerase chain reaction (PCR) was performed with an amplification volume of 50 μl. The program was set up as follows: 98 °C pre-denaturation for 2 min; 98 °C denaturation for 30 s, 65 °C annealing for 30 s, 72 °C extension for 1 min, total 10 cycles; 72 °C extension 10 min; 4 °C, hold. The product was purified with AMPure XP beads. (3) Amplified DNA was hybridized and placed at 65 °C for 16–24 h (4) After hybridization, stranded penicillin magnetic beads were applied for probe capture and PCR amplification. The amplification volume was 50 μl. The program was set up as follows: pre-denaturation at 98 °C for 2 min; denaturation at 98 °C for 30 s, annealing at 57 °C for 30 s, and extension at 72 °C for 1 min; 12 cycles; 72 °C extension for 10 min; 4 °C, hold. (5) AMPure XP beads were used for purification, Aglinet 2100 was used for quality control. The fragment size was about 250bp-350bp, and library preparation was completed.

Finally, Nextseq 500 (Illumina) was applied for PE75 sequencing.

### 2.3 Screening and functional analysis of candidate pathogenic genes

Trimmatomatic was used to remove the original sequencing connector and low-quality sequences. Filtered sequences were aligned to Human genome HG19 using BWA. Sequences were deduplicated with Picard. Using GATK software, single nucleotide variation (SNV) and indel mutation (In/Del) were analyzed and filtered. The called variants were annotated with ANNOVAR. Loci with a minor allele frequency (MAF) < 1 % in 1000 Genomes Project (1000G), Exome Aggregation Consortium (ExAC), and ESP6500 databases were screened. Furtherly, sites with non-synonymous variants were selected. SIFT, Polyphen2_HDIV, LRT, MutationTaster and PROVEAN were used to predict potential functions of selected variants. When an individual variant site was indicated to be harmful according to the three databases, its corresponding gene was classified as a “high-risk pathogenic variant”.

GeneCards database was searched for CD-related genes and those with association of >20 % were selected as “known CD pathogenic genes”. Colon tissue specific genes were searched in the TiGER database. Those genes overlapped between “high-risk pathogenic variants” and “known CD pathogenic genes” were selected. The protein-protein interaction network was constructed with Cytoscape3.6. The genes with protein-protein interactions were listed as candidate pathogenic genes.

KOBAS was used for signal enrichment analysis of candidate pathogenic genes. Swiss-model was used to predict the 3D structure of candidate pathogenic gene encoding protein. The SwissPDB viewer was used to identify structural changes before and after a mutation was introduced and to estimate alterations in force fields. InterProScan was used to predict protein domains.

### 2.4 Sanger sequencing

Based on the hG19 sequence of reference human genome, primers on both sides of candidate pathogenic gene loci were designed. The detailed information of primers for candidate pathogenic gene is shown in Table 1. Primers were synthesized by Sangon Biotech (Shanghai). Loci were amplified by PCR and sent to Sangon Biotech for Sanger sequencing.

**Table 1:** Primers used in PCR for amplification of candidate pathogenic genes.

Gene	Primer sequence (forward/reverse)	Product size (bp)
*NOD2*	PF 5′- CTTCAATTGTGGCAGGCCAGG -3′ PR 5′- CAATGTCACCCACAGAGTTGTAGTC -3′	530
*IL1*0RA	PF 5′- AATGAGCTGCCGTGGACTAGA -3′ PR 5′- AAAGTCCTGCCCTGTTTGGG -3′	480
*COL7A1*	PF 5′- GGCAGAAAGGTGTGTCTGGG -3′ PR 5′- CATTCAGTGGGAACAGTGGGGAG -3′	530
*PLA2G6*	PF 5′- CAACACGCACACCCTGAGAT -3′ PR 5′- TCCCCACTCATGCACACTTAGA -3′	480

## 3 Results

### 3.1 Identification and functional analysis of candidate pathogenic genes

The average whole exome sequencing depth of the target capture region from the child and her parents was 134 × , 89 × and 75 × , respectively. The sequencing coverage was more than 99.9 %. A total of 131169 variant loci were detected in the patient, including 12200 non-synonymous variant loci, as well as insertion/deletion loci in exons and variable splicing regions. Using 1000G database, ExAC database and Esp6500 database, the loci with a MAF>1 % were removed. The remaining 676 variant loci were selected. After predicting harmful variant sites, a total of 128 pathogenicity sites were identified, located on 124 genes (see Supplementary Table 1).

GeneCards obtained “known CD-related genes”. Totally, 375 genes with disease correlation >20 % were identified (see Supplementary Table 2). Five genes of the 124 high-risk pathogenic genes carried by the patient were associated with CD: *NOD2*, *IL1*0RA, *PRODH*, *PLA2G6*, and *COL7A1*. A total of 199 genes specifically expressed in normal colon were queried by TiGER (see Supplementary Table 3). Fig. 1 shows through protein-protein interaction analysis, *NOD2*, *IL1*0RA, *PLA2G6* and *COL7A1* genes were predicted to interact with colon-specific genes.

**Fig. 1:**
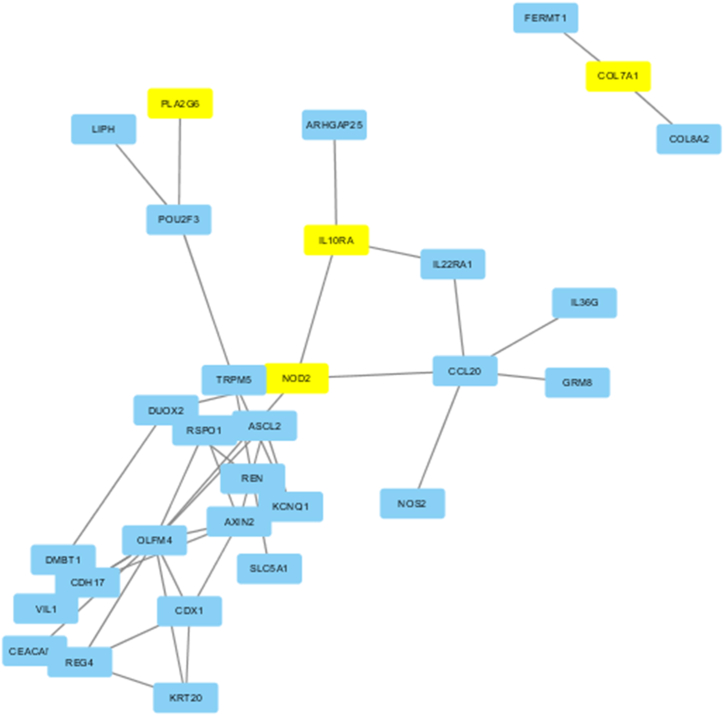
Diagram of interaction between candidate pathogenic genes and colon-specific gene proteins.

**Fig. 2:**
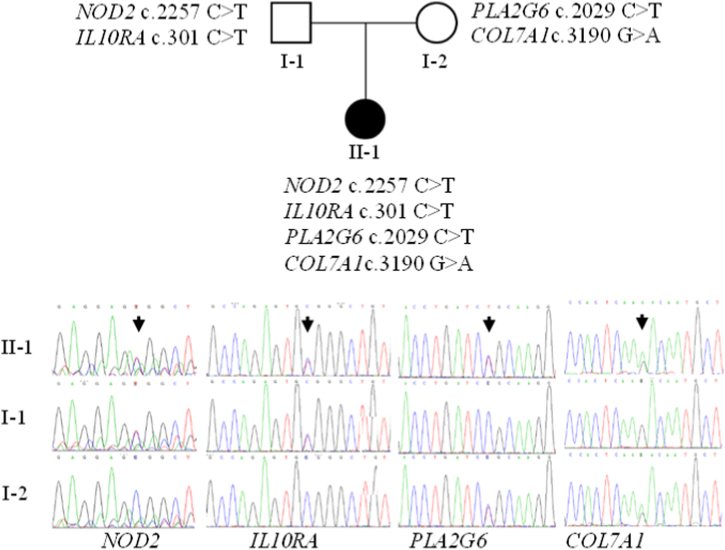
Genetic patterns and sequencing peaks of candidate pathogenic genes.

Fig. 2 depicts the heterozygous variants of *NOD2* c. 2257 C > T, *IL1*0RA c. 301 C > T, *PLA2G6* c. 2029 C > T and *COL7A1* c. 3190 G > A were carried by the patient. Her father carried heterozygous variants of *NOD2* c.2257 C > T and *IL1*0RA c.301 C > T, while her mother carried heterozygous variants of *PLA2G6* c.2029 C > T and *COL7A1* c.3190 G > A, as shown in Fig. 2.

Based on analysis of gene functions, *NOD2* might activate MAPK pathway and NF-κB pathway, and promote the transcriptional expression of immune-related genes, which in turn participate in the gastrointestinal immune response. IL10RA is a cell surface receptor for the cytokine IL10. IL10RA activates the JAK-STAT pathway, which promotes the expression of anti-inflammatory genes to restrict excessive tissue destruction caused by inflammation. PLA2G6 is a non-calcium-dependent phospholipase, involved in phospholipid remodeling, cell membrane homeostasis and signal transduction. *COL7A1* encodes α1 chain of collagen type VII that interacts with extracellular matrix to form anchored fibrils. These fibrils contribute to the formation and adhesion of epithelial basement membranes. Based on pathway analysis, these four genes were involved Cytokine-Cytokine receptor interaction, TNF signaling pathway, and JAK-STAT signaling pathway. Variants of *NOD2*, *IL1*0RA, *PLA2G6* and *COL7A1* might cause excessive intestinal inflammatory response and damage to the intestinal wall.

### 3.2 Protein structure prediction and functional analysis

**Fig. 3:**
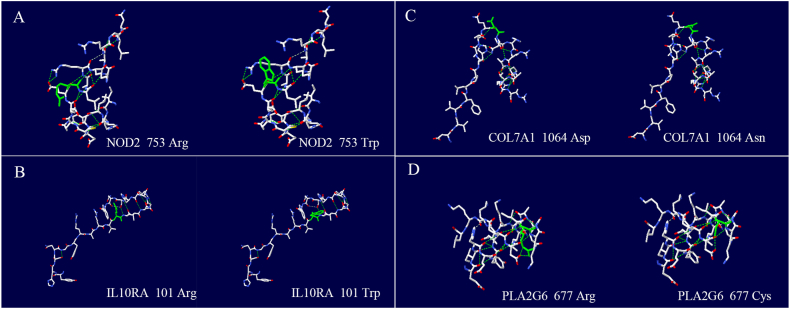
Schematic diagrams of 3D structures of candidate proteins. (A–D): The changes in protein structures attributed to NOD2 R753W, IL10RA R101W, PLA2G6 R677C and COL7A1 D1064 N. The green solid line represents the amino acids at the variant site; the green dotted line represents hydrogen bonds. (For interpretation of the references to color in this figure legend, the reader is referred to the Web version of this article.)

These variants in coding regions of genes might change proteins’ structures, functions and signal transduction, leading to biological dysfunction. Fig. 3A–D shows the changes of protein structure before and after NOD2 R753W, IL10RA R101W, COL7A1 D1064 N and PLA2G6 R677C mutations, respectively.

**Fig. 4:**
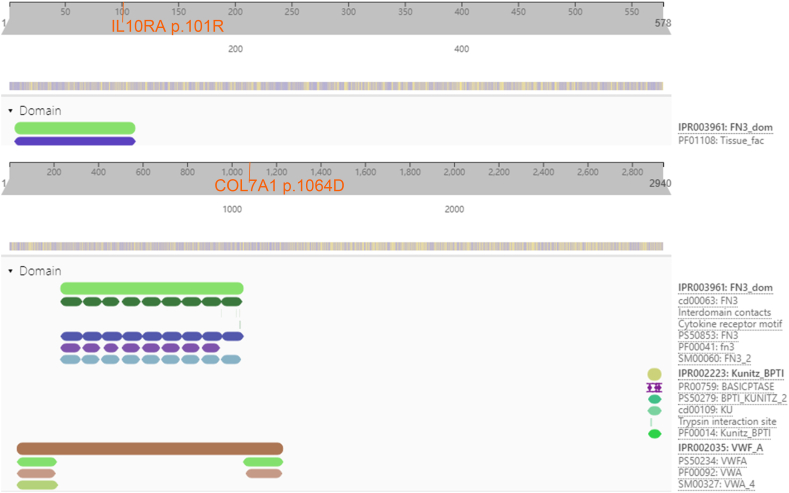
Schematic diagrams of predicted domains of IL10RA and COL7A1 proteins.

Fig. 4, describe the 101th amino acid of IL10RA is located on the FN3 domain, while the 1064th amino acid of COL7A1 is located on VWF-A domain. The 753rd amino acid of NOD2 is located on HD2 domain, the 677th amino acid of PLA2G6 is not located on PNPLA domain (see Supplementary Fig. 1). FN3 domain is involved in coagulation, inflammation, as well as cell adhesion and migration [21,22]. The VWF-A domain is involved in cell adhesion, migration, pattern formation and signal transduction [23,24]. The ARG to TRP mutation on 101th amino acid of IL10RA might result in overactivated inflammatory response. The ASP to ASN mutation on 1064th amino acid of COL7A1 might impair the intestinal wall barrier function.

## 4 Disscusion

In this study, whole exome sequencing was performed on the core family of a patient with CD at birth. Data analysis found that the patient carried four mutated genes: *NOD2* C.2257 c > T, *IL1*0RA C.301 C > T, *PLA2G6* C.2029 c > T, *COL7A1* C.3190 G > A.

*NOD2* was discovered as a CD-related gene. NOD2 participates in gastrointestinal immune response, activates MAPK and NF-κB signaling pathways, and upregulates immune-related genes [25]. IL10RA inhibits the synthesis of pro-inflammatory cytokines [26]. *IL-*10RA gene variant was found in 7 of the 14 children with CD-onset under age 1 [27]. *NOD2* c.2257 C > T and *IL1*0RA c.301 C > T variants carried by the patient were inherited from her father. *NOD2* and *IL1*0RA variant correlates with dysregulated Cytokine-Cytokine receptor interaction and TNF signaling pathway, continuous expression of inflammatory cytokines, leads to excessive inflammatory response. When the excessive inflammatory response cannot be alleviated, intestinal wall tissue would be injured. Paneth cells produce less defensin, leading to cell death.

*PLA2G6* encodes phospholipase A2 protein, which is involved in phospholipid remodeling, cell membrane homeostasis and signal transduction. COL7A1 acts as an anchor for the protofilament between the epithelium and the stroma, which contributes to the formation and adhesion of basement membrane [28]. The frequency of *COL7A1* rs3197999 allele was significantly higher in IBD patients than in healthy subjects [29]. *PLA2G6* c.2029 C > T and *COL7A1* c.3190 G > A variants were inherited from her mother. PLA2G6 variant might result in disorder of cell surface receptor signal transduction, and affect cell proliferation and differentiation. COL7A1 variant might cause intestinal wall barrier function and repair capability would be reduced or impaired.

Notably, her parents reported a family history of immune diseases of varying degrees. Her father suffered from mild asthma as a child, and recovered after puberty. Her mother suffered from mild dermatitis. Gene Cards obtained “known immunodeficiency related genes”. Totally, 145 genes with disease correlation >20 % were identified (see Supplementary Table 4). *TNFRSF13B* is the gene that overlaps in the high-risk pathogenic gene and the immunodeficiency gene. *TNFRSF13B*, induces activation of the transcription factors AP1 and NF-κB, and plays a crucial role in humoral immunity by interacting with a TNF ligand. The patient carries variant gene and exhibited severe CD phenotype. Her parents carry variant gene, without clinical manifestations of intestinal inflammation and CD. We need further experiments to verify the function of single gene variant. The synergistic effects of variants of *NOD2*, *IL1*0RA, *PLA2G6* and *COL7A1*, also needs additional experiments verification.

## Funding

This study received no funding.

## Ethics declaration

This study was approved by the Ethics Committee of Liaocheng People's Hospital. All subjects had signed informed consent. Written informed consent of children was provided by their legal guardians.

## Data availability statement

The data that support the results have been deposited into a publicly available repository: The Genome Variation Map (GVM) in Big Data Center, Beijing Institute of Genomics (BIG), Chinese Academy of Science, the accession number GVM000319, accessible at http://bigd.big.ac.cn/gvm/getProjectDetail?project=GVM000319. All other relevant data from this study will be available from the corresponding authors upon reasonable request.

## CRediT authorship contribution statement

**Qiang Zhang:** Conceptualization, Formal analysis, Investigation, Methodology, Validation, Visualization, Writing – original draft, Writing – review & editing. **Xizi Wang:** Conceptualization, Data curation, Formal analysis, Methodology, Software, Visualization, Writing – original draft. **Juan Zheng:** Investigation, Methodology, Validation. **Qiang Lü:** Formal analysis, Methodology, Software, Visualization. **Rongrong Li:** Investigation, Methodology, Validation. **Xiaodong Jia:** Data curation, Methodology, Resources, Software. **Mingliang Gu:** Conceptualization, Data curation, Formal analysis, Methodology, Project administration, Resources, Supervision, Writing – review & editing.

## Declaration of competing interest

The authors declare that they have no known competing financial interests or personal relationships that could have appeared to influence the work reported in this paper.

## Figures and Tables

**figs1:**
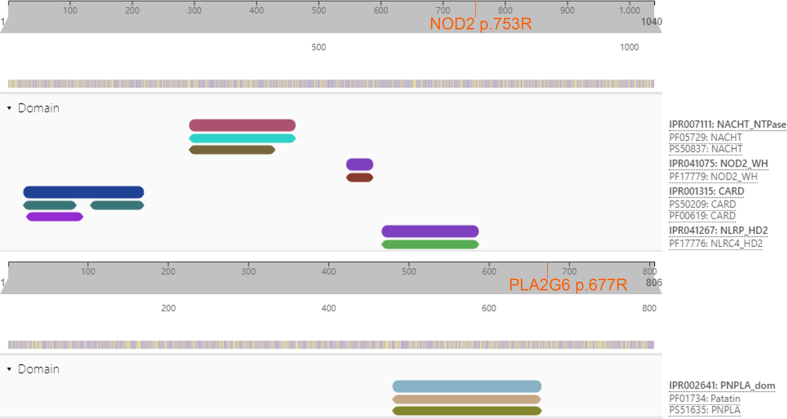
Schematic diagrams of predicted domains of NOD2 and PLA2G6 proteins.
